# Tailoring the Size and Shape of ZnO Nanoparticles for Enhanced Performance of OLED Device

**DOI:** 10.3390/nano13212816

**Published:** 2023-10-24

**Authors:** Nikita Khairnar, Hyukmin Kwon, Sangwook Park, Hayoon Lee, Jongwook Park

**Affiliations:** Integrated Engineering, Department of Chemical Engineering, Kyung Hee University, Yongin-si 17104, Republic of Korea; nikita0120@khu.ac.kr (N.K.); hm531@khu.ac.kr (H.K.); pswook@khu.ac.kr (S.P.); kssarang1@khu.ac.kr (H.L.)

**Keywords:** OLEDs, metal oxide, ZnO nanoparticles, EIL, ETL

## Abstract

We synthesized zinc oxide nanoparticles (ZnO NPs) by meticulously controlling both temperature and reaction times, allowing us to fine-tune their crystalline properties, morphology, and particle dimensions. This analysis confirmed the existence of a mixture of rod and sphere shapes (ZnO-I), including rod-shaped NPs with an average size of 14.8 nm × 5.2 nm and spherical NPs with an average diameter of 5.27 nm. We subsequently incorporated these synthesized ZnO NPs into organic light-emitting diode (OLED) devices for red, green, and blue colors, utilizing them as the electron injection layer through a solution-based process. The green OLED device using ZnO-I exhibited a promising current efficiency of 4.02 cd/A and an external quantum efficiency of 1.47%.

## 1. Introduction

Metal oxides have garnered significant attention as semiconducting materials for various optoelectronic applications, including transistors, photodetectors, lasers, solar cells, and light-emitting diodes (LEDs) [1,2,3,4,5]. These materials exhibit outstanding electronic properties, such as suitable bandgaps, low trap densities, tunable emissions, and high absorption coefficients, making them promising candidates for optoelectronic applications. Among these metal oxides, those based on colloidal solutions, such as zinc oxide (ZnO), titanium dioxide (TiO_2_), and tin oxide (SnO_2_), find widespread use as electron-transporting layers (ETLs) in devices employing solution processes [6,7,8]. In particular, ZnO stands out as a prime choice for the electron-injection layer (EIL) and ETL among metal oxides in solution process-based inverted organic light-emitting diodes (OLEDs) [9,10]. Furthermore, ZnO NPs are often used in OLEDs with the conventional device configuration [11,12,13]. This preference arises from ZnO’s inherent high electron mobility, proper exciton binding energy, and ease of fabrication at room temperature. Additionally, ZnO’s work function value of −4.1 eV closely matches that of indium tin oxide (ITO), making it suitable for inverted device configurations [14]. However, achieving the synthesis of ZnO nanoparticles with small size and uniformity remains a challenging task. Moreover, ZnO’s relatively large energy band gap of 3.3 eV (with HOMO −7.4 eV/LUMO −4.1 eV) results in a substantial energy barrier with adjacent organic layers, leading to elevated driving voltages and difficulties in electron injection [15,16].

In this study, we present a comprehensive investigation focusing on the impact of reaction parameters on the synthesis of ZnO NPs, with specific emphasis on controlling their size and shape. We manipulated reaction time and temperature to achieve the desired nanoparticle sizes and a rod-like shape for ZnO NPs. We investigated the optical properties, morphology, and nanostructure of the synthesized ZnO NPs. Furthermore, we utilized the synthesized ZnO NPs as EILs in the fabrication of inverted OLED devices in blue, green, and red colors, employing a hybrid process that combines spin coating of the synthesized ZnO with the evaporation method. The inverted device structure offers improved durability compared to conventional configurations since the EIL and ETL, which are sensitive to oxygen and humidity, can be shielded within other layers and metals [17,18]. Additionally, we introduce a polyethyleneimine (PEI) layer to mitigate the energy barrier between ZnO and the emitting layer (EML) [19,20]. This work demonstrates the feasibility of inverted white OLEDs (WOLEDs) utilizing ZnO, as we successfully manufacture OLED devices in all three primary colors (blue, green, and red) with the synthesized ZnO.

## 2. Materials and Methods

### 2.1. General Information

Reagents and solvents were purchased as reagent grade and used without further purification. The chemical structure of the compounds was obtained with Fourier-transform infrared spectroscopy (FT-IR) using a FT-IR 4100 (JASCO, Tokyo, Japan). Field emission scanning electron microscopy (FESEM) images of the ZnO layers were obtained using a JSM-7900F instrument (JEOL, Tokyo, Japan). Transmission electron microscopy (TEM) images of the ZnO NPs were obtained using a Tecnai G2 F30 S-Twin (FEI, Hillsborough, OR, USA). The particle sizes were determined using ImageJ software (version 1.54d). ImageJ is a Java-based, multithreaded, freely available, open-source, platform-independent, and public domain program for image processing and analysis. It was developed at the National Institutes of Health in the USA. X-ray diffraction (XRD) patterns of the ZnO layer were recorded using a SmartLab 9 kW diffractometer (Rigaku, Tokyo, Japan), target material: Cu, λ = 0.15406 nm). Optical ultraviolet visible (UV-Vis) absorption spectra were obtained using a UV-1900i UV/Vis/NIR spectrometer (Shimadzu, Kyoto, Japan). A Perkin-Elmer LS55 (Xenon flash tube) luminescence spectrometer was used to analyze photoluminescence (PL) spectroscopy. Atomic force microscopy (AFM) images were obtained using a D3100 microscope (Veeco, Plainview, NY, USA). The I–V–L characteristics, EL spectra, and CIE color coordinates of the solution-process OLEDs were measured using a Keithley 2400 SMU sourcemeter and CS-1000A spectroradiometer (Konica-Minolta, Tokyo, Japan) at room temperature under ambient conditions.

### 2.2. Synthesis of ZnO

By referring Figure S1, 1.678 g of zinc acetate was dissolved in 84 mL of methanol under nitrogen, and then 250 µL of deionized water was added. The solution was then heated at 60 °C until the zinc acetate was completely dissolved. A total of 0.98 g of potassium hydroxide in 46 mL of methanol was added slowly to the zinc acetate solution over a period of 15 min. The solution was stirred at 60 °C for 150 min and then allowed to cool to room temperature for 90 min. The methanol was evaporated from the mixture to reduce its volume to 20 mL. The concentrated mixture was then refluxed for 18 h to obtain the sphere (ZnO-S) and 12 h for mixing the rod and sphere shapes (ZnO-I). The supernatant was carefully eliminated, and the mixture was washed with 100 mL of methanol three times. Following this, the mixture was left overnight, and the supernatant was again slowly removed until the volume of the ZnO mixture was reduced to 3–5 mL. In addition, the residual solution was evaporated, and the resulting powder was then subjected to further analyses [21].

### 2.3. Device Fabrication

ITO glass substrates (25 mm × 25 mm) were ultrasonicated sequentially in acetone, ethanol, deionized water, and isopropyl alcohol and then dried in an oven at 80 °C. The substrates were further cleaned in a UV–ozone cleaner for 10 min. The ZnO solution (4 mg/1 mL in methanol) was spin-coated at 2000 rpm for 60 s and then annealed at 80 °C for 15 min. The other spin-coated processes were as follows. PEI (0.4 wt% in 2-metoxyehtanol)—5000 rpm, 60 s, and annealed at 140 °C for 20 min; poly[2-methoxy-5-(2′-ethylhexyloxy)-1,4-phenylene vinylene] (MEH-PPV) (4 mg/1 mL in chlorobenzene)—2000 rpm, 50 s, and annealed at 140 °C for 10 min; poly(9,9-dioctylfluorene-alt-benzothiadiazole) (F8BT) (12 mg/1 mL in chlorobenzene)—2500 rpm, 50 s, and annealed at 140 °C for 10 min; bathophenanthroline (Bphen) (3 mg/3 mL in ethanol)—2000 rpm, 30 s, and annealed at 80 °C for 15 min; 9-(1-naphthyl)-10-(2-naphthyl) anthracene (α,β-ADN): 5 wt% 3Me-1Bu-TPPDA (α,β-ADN 19 mg and 3Me-1Bu-TPPDA 1 mg/1 mL in chlorobenzene)—2500 rpm, 50 s, and annealed at 140 °C for 5 min. After cooling to room temperature, the prepared substrates were transferred to a thermal evaporation chamber. The chamber was evacuated to 2.0 × 10^−5^ torr, and 1,1-Bis[(di-4-tolylamino)phenyl]cyclohexane (TAPC) (Lumtec, Taiwan), MoO_3_, and Al were sequentially evaporated.

## 3. Results and Discussion

### 3.1. Synthetic Design of ZnO NPs

The synthesis method is described in detail in Section 2.2 and Figure S1. It is noteworthy that metal oxides can undergo changes in size and shape in response to variations in temperature and time due to a phenomenon known as thermal oxidation. This phenomenon is rooted in the capability of metal oxides to undergo physical changes as they are heated, leading to alterations to their structure and properties. At elevated temperatures, metal oxides can undergo diffusion, wherein molecules migrate within the material, leading to the formation of new structures [22]. We investigated the newly synthesized ZnO NPs by applying reaction times of 18 h (ZnO-S) and 12 h (ZnO-I) based on this principle. It was also confirmed that controlling the temperature and reaction time was key to determining the ZnO size and shape.

### 3.2. Characterization of ZnO

**Figure 1:**
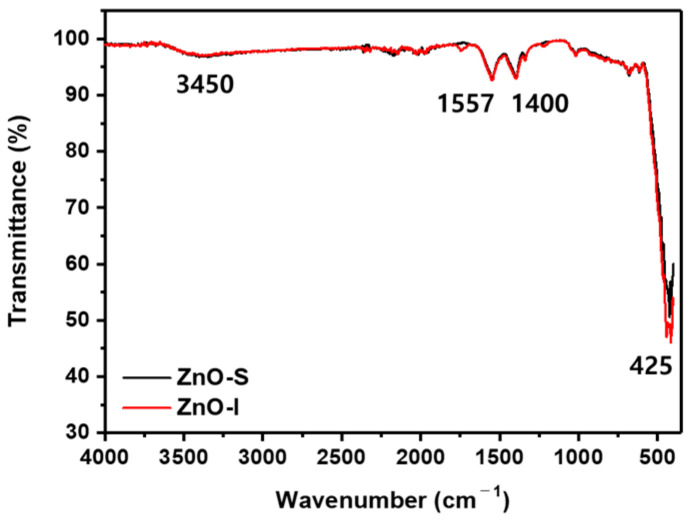
FT-IR spectra of ZnO-S and ZnO-I.

We performed qualitative chemical analysis using FT-IR to confirm the presence of impurities in the synthesized ZnO NPs and to identify characteristic peaks of ZnO (Figure 1). The FT-IR spectra of the synthesized ZnO NPs exhibit the typical peaks of ZnO. Both ZnO-S and ZnO-I show strong absorption peaks in the range of 400–480 cm^−1^, corresponding to the metal oxide stretching vibration of Zn-O bonds. The weaker intensity peaks at 1400 and 1557 cm^−1^ correspond to the stretching vibration of the C-O bonds in methanol, which was used during synthesis and purification. The small and broad peak in the range of 3250–3500 cm^−1^ is associated with the O-H stretching vibration of methanol. Since no organic surfactants were used during the synthesis process, there were no impurity peaks observed. This indicates that high-purity ZnO NPs were successfully synthesized [23].

**Figure 2:**
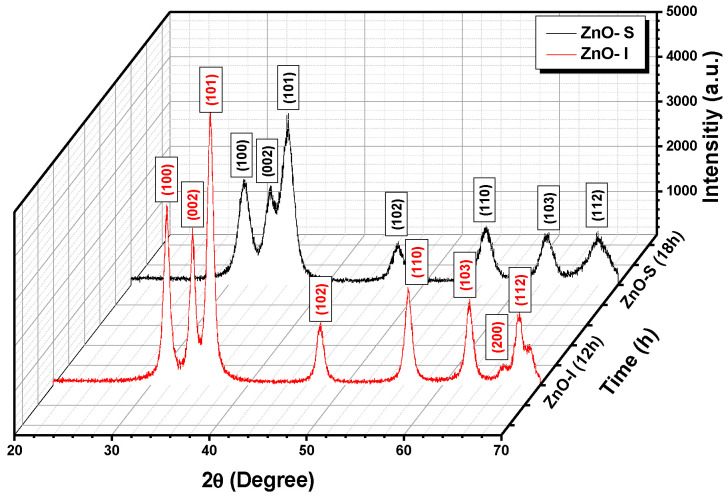
XRD patterns of ZnO-S and ZnO-I.

Figure 2 presents typical XRD patterns of the ZnO NPs within the 2θ range from 20° to 70°. The volume and crystallite size data are available in Tables S1 and S2. The XRD results strongly suggest the formation of a hexagonal wurtzite structure of ZnO, consistent with the reference pattern (JCPDS No. 01-075-0576) [24]. The indexed peaks correspond to the (100), (002), (101), (102), (110), (103), and (112) planes. Particularly noteworthy is the (002) reflection peak at 34.3°, positioned between the (100) reflection at 31.7° and the (101) reflection at 36.1°, observed for both ZnO-S and ZnO-I. This relatively sharp (002) reflection peak signifies a rod-like domain, corroborating the TEM observations [21,24].

**Figure 3:**
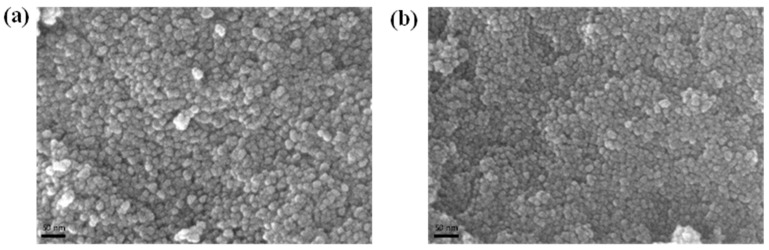
FESEM analysis at different magnifications for (**a**) ZnO-S and (**b**) ZnO-I.

To assess the uniformity of the coated ZnO NP films, FESEM analysis was conducted (Figure 3). Interestingly, no pinholes, which can act as carrier traps leading to reduced device efficiency, were detected in the SEM images for both ZnO-S and ZnO-I. An absence of defects and a uniform surface condition not only mitigate overcurrent and leakage current during the OLED device operation but also enhance the stability of the light-emitting layer, ultimately contributing to stable device performance [25,26].

**Figure 4:**
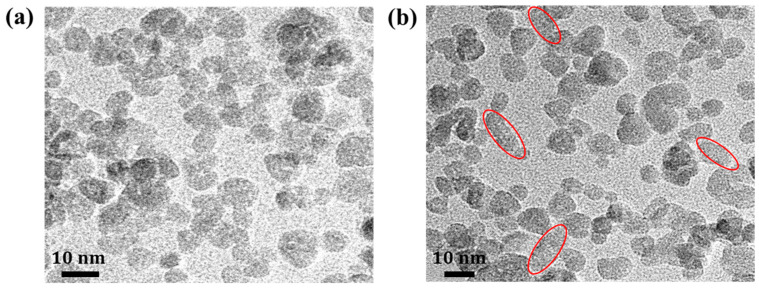
TEM analysis of ZnO NPs (**a**) ZnO-S and (**b**) ZnO-I.

TEM analysis of the ZnO powder revealed distinctive surface morphologies (Figure 4). ZnO-S primarily exhibited a spherical shape, while ZnO-I showed a mixture of rod-like and spherical forms, as marked by the red oval shapes. These rod-shaped structures are present at a 12 h reaction time but absent at 18 h. Although the synthesis conditions for ZnO-S and ZnO-I are almost identical, with the only difference being the reaction time, shape variations associated with this difference have been reported in other studies [21]. Additionally, it was confirmed that when the reaction time is shorter than 12 h, there are fewer rod-like shapes, and the device performance is suboptimal, demonstrating that the 12 h reaction time is the optimal duration. The average diameter of the spherical ZnO particles was measured at 5.62 nm for ZnO-S and 5.27 nm for ZnO-I. In the case of ZnO-I, the rod-shaped particles exhibited dimensions of 14.8 nm × 5.2 nm. It is known that as the shape of ZnO NPs approaches the rod-like form, it contributes to the formation of a denser film with a larger contact area. This difference in ZnO morphologies is expected to have a significant impact on the movement of electron carriers, ultimately influencing the OLED device’s performance. Therefore, when using metal oxide as a carrier transporting layer, it is advisable to conduct preliminary experiments to optimize the shape and size of the crystal for each metal oxide and apply the findings to the device.

**Figure 5:**
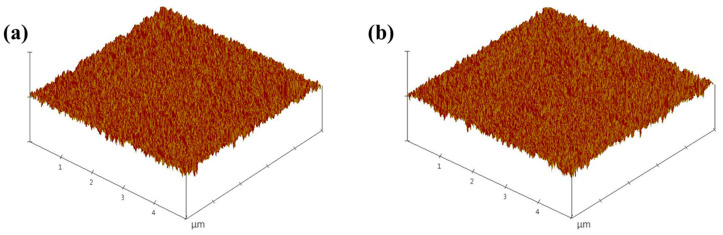
AFM images of (**a**) ZnO-S and (**b**) ZnO-I.

Surface morphology is important for solution-processed OLED device fabrication. AFM was employed to analyze the surface properties of spin-coated films using the synthesized ZnO NPs (Figure 5). The root mean square (RMS) roughness values for ZnO-S and ZnO-I were 1.45 nm and 1.43 nm, respectively, indicating that both films had smooth surfaces. These smooth films are beneficial for enhancing device efficiency due to efficient electron carrier mobility [27,28].

These comprehensive XRD, FESEM, TEM, and AFM characterizations provide important criteria into the size, shape, and uniformity of the synthesized ZnO NPs, setting the stage for further analysis of their impact on OLED device performance (Table 1).

### 3.3. Photophysical Properties of ZnO

**Figure 6:**
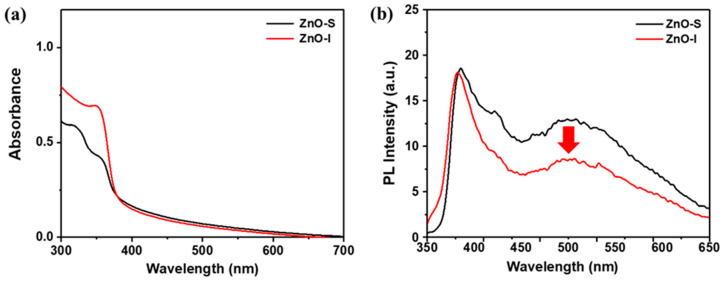
(**a**) Absorption spectra of ZnO-S and I; (**b**) PL spectra of ZnO-S and I.

The optical properties of ZnO-S and ZnO-I were investigated using UV-Vis absorption and PL (Figure 6). In order to be suitable as an EIL for inverted OLED devices, they should exhibit minimal absorption in the 400–800 nm range, ensuring high light transmittance towards the ITO substrate. Both ZnO-S and ZnO-I exhibited maximum absorption peaks at 358 nm and 354 nm, respectively. Both the absorption spectra of ZnO-S and ZnO-I exhibit smoother absorbance in the range of 380 nm to 700 nm. This may be attributed to peaks resulting from the reflection and scattering of NPs within the partially dissolved ZnO suspension. The optical band gaps, estimated from the absorption edge, were found to be 3.20 eV for ZnO-S and 3.24 eV for ZnO-I. In terms of PL spectra, variations in the level of crystallinity were observed. While both ZnO-S and ZnO-I exhibited similar shapes and emission peaks at around 380 and 500 nm, which are attributed to the transition of the ZnO crystal and the trap state emission, respectively, ZnO-I exhibited lower PL intensity at 500 nm [29,30]. This might mean that ZnO-I, which possesses a more crystalline structure with fewer trap-state emissions compared to ZnO-S and is characterized by its lower crystallinity and more trap-state emissions, is expected to enhance the efficiency of OLED devices.

### 3.4. Electroluminescence (EL) Properties of OLED Devices

**Figure 7:**
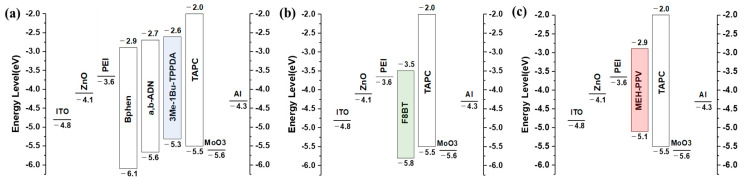
Band diagrams of each device: (**a**) blue, (**b**) green, and (**c**) red.

Inverted OLED devices of blue, green, and red color were fabricated using a hybrid process involving the spin coating of the synthesized ZnO and evaporation methods. The device configurations for each primary color were as follows: blue (B)—ITO/ZnO (solution, 30 nm)/PEI (solution, 10 nm)/Bphen (solution, 20 nm)/α,β-ADN: 5 wt% 3Me-1Bu-TPPDA (solution, 30 nm)/TAPC (evaporation, 20 nm)/MoO_3_ (evaporation, 5 nm)/Al (evaporation, 200 nm); green (G)—ITO/ZnO (solution, 30 nm)/PEI (solution, 5 nm)/F8BT (solution, 80 nm)/TAPC (evaporation, 20 nm)/MoO3 (evaporation, 5 nm)/Al (evaporation, 200 nm); red (R)—ITO/ZnO (solution, 30 nm)/PEI (solution, 10 nm)/MEH-PPV (solution, 40 nm)/TAPC (evaporation, 20 nm)/MoO_3_ (evaporation, 5 nm)/Al (evaporation, 200 nm). ZnO was used as the EIL, and PEI served as the ETL and interlayer. PEI improves the interfacial properties between the inorganic material ZnO and the EML [31]. TAPC and MoO_3_ were used as the hole-transporting layer (HTL) and hole-injection layer (HIL), respectively. MEH-PPV and F8BT were utilized as red and green polymers, and 3Me-1Bu-TPPDA, a blue fluorescent molecule, was employed for the first time in a solution process [32]. Notably, achieving stable blue devices is relatively challenging due to the wider band gap of blue EML compared to green and red colors (Figure 7).

**Figure 8:**
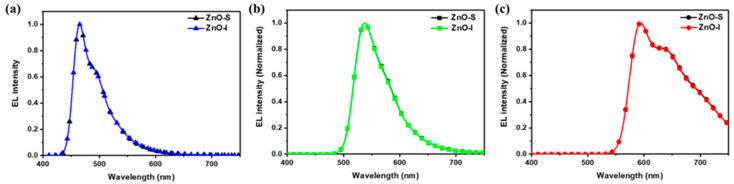
EL spectra of devices: (**a**) blue, (**b**) green, (**c**) red.

The EL performance for each device is shown in Figures S2–S4, and Table 2 summarizes the efficiency of the fabricated inverted OLED device. The results of the OLED device performance in this study are quite significant and provide valuable insights into the efficiency improvements achieved by using different ZnO NPs shapes. The OLED devices achieved emission spectra with peak wavelengths of 465 nm (blue), 538 nm (green), and 593 nm (red). The devices, with Commission Internationale de l’Éclairage (CIE) coordinates of (0.156, 0.243), (0.378, 0.600), and (0.606, 0.393) for blue, green, and red, respectively, were also matched with each EML emission, suggesting effective recombination zones within the EML (Figure 8).

We have included the operating voltages for red, green, and blue color devices, all normalized to a current density of 10 mA/cm^2^ in Table 2. All devices using ZnO-I exhibited lower operating voltages than those using ZnO-S. This can be explained by the rod-like ZnO NPs reducing electron trap states in their crystals and improving the electron transport properties. Upon reviewing this data, it is evident that green OLEDs exhibit a relatively lower operating voltage due to a small energy barrier between PEI (LUMO: −3.6 eV) and the emissive layer F8BT (LUMO: −3.5 eV). In contrast, red OLEDs face a relatively larger operating voltage, attributed to the relatively large energy barrier between PEI (LUMO: −3.6 eV) and the emissive layer MEH-PPV (LUMO: −2.9 eV) [33]. In the case of blue devices, the LUMO energy barrier between PEI (LUMO: −3.6 eV) and BPhen (LUMO: −2.9 eV) was similar to that in red devices. However, these two devices still exhibited an operating voltage difference of approximately 1.5 V. In our future research, we plan to address this issue through device optimization and interface analysis. As a result, the power efficiency (PE) decreased in the following order: green, blue, and red. The PE for the blue devices using ZnO-S and ZnO-I was 0.21 and 0.28 lm/W, respectively. For the green devices, it was 1.26 and 2.01 lm/W, and for the red devices, it was 0.08 and 0.12 lm/W, respectively. This resulted in a 33% increase for blue, 60% for green, and 50% for red in the PE values between the devices using ZnO-S and ZnO-I. In the case of the three color devices, the ZnO-I devices with a rod-like shape had higher efficiency compared to the ZnO-S devices, due to the larger surface contact of the adjacent ZnO NPs, which facilitated smooth carrier migration. The ZnO-S device for blue had a luminance efficiency (LE) of 0.44 cd/A and an external quantum efficiency (EQE) of 0.26%, while the ZnO-I device for blue achieved an LE of 0.63 cd/A and an EQE of 0.37%. For the green devices, the ZnO-S device showed an LE of 3.06 cd/A and an EQE of 0.83%, while the ZnO-I device had the best EL performance among the three color devices, with an LE of 4.02 cd/A and an EQE of 1.47%. The ZnO-S device for red exhibited an LE of 0.26 cd/A and an EQE of 0.19%, but the ZnO-I device for red showed an LE of 0.36 cd/A and an EQE of 0.27%. These results represent improved efficiency values of 44% for blue, 31% for green, and 38% for red in each device. To ensure the reliability of our devices, we measured the lifetimes of the devices and tested multiple instances of the same device, and we have confirmed that the EQE efficiency can be achieved with an error rate of less than 7% (Figures S5 and S6). The overall EL efficiencies of the three devices are relatively low; however, there is potential for further improvement in efficiency by using more efficient emitters in the red, green, and blue colors. Additionally, addressing the relatively large energy barriers for holes and electrons within the device configuration could result in even better performance. This might entail selecting different carrier transport layers and optimizing layer thicknesses in the future.

## 4. Conclusions

We successfully synthesized rod-like and spherical ZnO NPs and found a significant influence of reaction parameters, particularly time and temperature, on the size and morphology of the resulting ZnO nanoparticles. The ZnO-I devices exhibited superior overall efficiency when compared to their spherical shape. Specifically, the green ZnO-I device showed an LE of 4.02 cd/A, an EQE of 1.47%, and a CIE color coordinate of (0.378, 0.600). Our results confirm that ZnO can serve as an EIL for RGB color devices, offering the potential for the fabrication of high-performance inverted-type white OLEDs through a solution-based process. Also, these insights into tailoring the properties of ZnO NPs pave the way for future applications in large-area OLED production using solution processes.

## Figures and Tables

**Table 1:** Characterization of the ZnO NPs.

Sample	λ_Abs_ (nm)	λ_PL_ (nm)	Band Gap ^a^ (eV)	Average Size (nm)		RMS ^d^ (nm)
				Sphere ^b^	Rod ^c^	
ZnO-S	358	385	3.20	5.62	-	1.45
ZnO-I	354	382	3.24	5.27	14.8 × 5.2	1.43

^a^ The optical bandgap was calculated according to the absorption edge; ^b^ the average diameter of the sphere-shaped ZnO was measured using TEM; ^c^ the average length and width size of the rod-like ZnO was measured using TEM; ^d^ the roughness was obtained from AFM.

**Table 2:** EL performances of the fabricated blue (B), green (G), and red (R) OLEDs.

@10 mA/cm^2^	V (V)	LE (cd/A)	PE (lm/W)	EQE (%)	CIE (x, y)	EL_max_ (nm)
ZnO-S (B)	8.19	0.44	0.21	0.26	(0.155, 0.242)	465
ZnO-I (B)	7.67	0.63	0.28	0.37	(0.156, 0.243)	465
ZnO-S (G)	7.60	3.06	1.26	0.83	(0.378, 0.600)	538
ZnO-I (G)	6.80	4.02	2.01	1.47	(0.378, 0.600)	538
ZnO-S (R)	9.77	0.26	0.08	0.19	(0.606, 0.393)	593
ZnO-I (R)	9.50	0.36	0.12	0.27	(0.606, 0.393)	593

## Data Availability

Not applicable.

## Supplementary Materials

The following supporting information can be downloaded at https://www.mdpi.com/article/10.3390/nano13212816/s1, Figure S1: The schematic synthesis method for ZnO NPs; Figure S2: EL performance for blue device: (a) current density (J)–voltage (V)–luminance (L) characteristics, (b) luminance efficiency (LE)–J curves, (c) external quantum efficiency (EQE)–J curves, (d) power efficiency (PE)–J curves; Figure S3: EL performance for green device (a) J–V–L characteristics, (b) LE–J curves, (c) EQE–J curves, (d) PE–J curves; Figure S4: EL performance for red device: (a) J–V–L characteristics, (b) LE–J curves, (c) EQE–J curves, (d) PE–J curves; Figure S5: Lifetime of devices (a) blue, (b) green, (c) red; Figure S6: EQE curves according to the current density: (a) blue, (b) green, (c) red; Table S1: Volume of unit cell of ZnO NPs; Table S2: Crystallite size of ZnO NPs. Reference [34] is cited in the supplementary materials.

## Sample Availability

Samples of the compounds are available from the authors.

## References

[1] Znaidi L., Illia G., Benyahia S., Sanchez C., Kanaev A.V. (2003). Oriented ZnO Thin Films Synthesis by Sol–Gel Process for Laser Application. Thin Solid Films.

[2] Liu K., Sakurai M., Aono M. (2010). ZnO-Based Ultraviolet Photodetectors. Sensors.

[3] Anta J.A., Guillén E., Tena-Zaera R. (2012). ZnO-Based Dye-Sensitized Solar Cells. J. Phys. Chem. C.

[4] Uthirakumar P., Kim H., Hong C.H. (2009). Zinc Oxide Nanostructures Derived from a Simple Solution Method for Solar Cells and LEDs. Chem. Eng. J..

[5] Liu C., Lee W., Shih T. (2012). Synthesis of ZnO Nanoparticles to Fabricate a Mask-Free Thin-Film Transistor by Inkjet Printing. J. Nanotechnol..

[6] Jeong K.W., Kim H.S., Yi G.R., Kim C.K. (2018). Enhancing the Electroluminescence of OLEDs by Using ZnO Nanoparticle Electron Transport Layers That Exhibit the Auger Electron Effect. Mol. Cryst. Liq. Cryst..

[7] Haque S.A., Koops S., Tokmoldin N., Durrant J.R., Huang J., Bradley D.D.C., Palomares E. (2007). A Multilayered Polymer Light-Emitting Diode Using a Nanocrystalline Metal-Oxide Film as a Charge-Injection Electrode. Adv. Mater..

[8] Wang H., Yu H., Xu W., Yuan Z., Yan Z., Wang C., Liu X., Fahlman M., Liu J.M., Liu X.K. (2018). Efficient Perovskite Light-Emitting Diodes Based on a Solution-Processed Tin Dioxide Electron Transport Layer. J. Mater. Chem. C.

[9] Dong D., Wang Y., Lian L., Feng D., Wang H., He G. (2017). Novel Solution-Processed ZnO-Based Electron Injection Layer for Organic Light-Emitting Diodes. Phys. Status Solidi A.

[10] Hwang H., Park H., Moon D. (2022). Highly Efficient Inverted Phosphorescent Organic Light-Emitting Devices with ZnO Nanoparticles Electron Injection Layer. Synth. Met..

[11] Qian L., Zheng Y., Choudhury K.R., Bera D., So F., Xue J., Holloway P.H. (2010). Electroluminescence from light-emitting polymer/ZnO nanoparticle heterojunctions at sub-bandgap voltages. Nano Today.

[12] Huang C.Y., Lai J.H. (2016). Efficient polymer light-emitting diodes with ZnO nanoparticles and interpretation of observed sub-bandgap turn-on phenomenon. Org. Electron..

[13] Huang C.Y., Cheng C.Y., Shih Y.S. (2017). All-solution-processed fluorene/dibenzothiophene-S,S-dioxide blue co-oligomer light-emitting diodes with an electron transporting PEI/ultrafine-ZnO-nanoparticle bilayer. RSC Adv..

[14] Höfle S., Schienle A., Bruns M., Lemmer U., Colsmann A. (2014). Enhanced Electron Injection into Inverted Polymer Light-Emitting Diodes by Combined Solution-Processed Zinc Oxide/Polyethylenimine Interlayers. Adv. Mater..

[15] Sasaki T., Hasegawa M., Inagaki K., Ito H., Suzuki K., Onno T., Morii K., Shimizu T., Fukagawa H. (2021). Unravelling the electron injection/transport mechanism in organic light-emitting diodes. Nat. Commun..

[16] Sasaki T., Onno T., Shimizu T., Fukagawa H. (2023). Effects of Energy-Level Alignment on Operating Voltages of Blue Organic Light-Emitting Diodes. Adv. Mater. Interfaces.

[17] Zhang M., Höfle S., Czolk J., Mertens A., Colsmann A. (2015). All-Solution Processed Transparent Organic Light Emitting Diodes. Nanoscale.

[18] Lee H., Park I., Kwak J., Yoon D.Y., Lee C. (2010). Improvement of Electron Injection in Inverted Bottom-Emission Blue Phosphorescent Organic Light Emitting Diodes Using Zinc Oxide Nanoparticles. Appl. Phys. Lett..

[19] Zhou T., Ling Z., Tang Z., Wang S., Guo K., Chen G., Cheng Z., Dai X., Gao H., Xu T. (2018). Efficient Solution-Processed Inverted Organic Light-Emitting Diodes by Using Polyethyleneimine as Interface Layer. Phys. Status Solidi A.

[20] Kim Y.H., Han T.H., Cho H., Min S.Y., Lee C.L., Lee T.W. (2014). Polyethylene Imine as an Ideal Interlayer for Highly Efficient Inverted Polymer Light-Emitting Diodes. Adv. Funct. Mater..

[21] Pu Y.J., Morishita N., Chiba T., Ohisa S., Igarashi M., Masuhara A., Kido J. (2015). Efficient Electron Injection by Size- and Shape-Controlled Zinc Oxide Nanoparticles in Organic Light-Emitting Devices. ACS Appl. Mater. Interfaces.

[22] Fang F., Zhao D.X., Zhang J.Y., Shen D.Z., Lu Y.M., Fan X.W., Li B.H., Wang X.H. (2008). The Influence of Growth Temperature on ZnO Nanowires. Mater. Lett..

[23] Abdelghani G.M., Ahmed A.B., Al-Zubaidi A.B. (2022). Synthesis, characterization, and the influence of energy of irradiation on optical properties of ZnO nanostructures. Sci. Rep..

[24] Wilken S., Scheunemann D., Wilkens V., Parisi J., Borchert H. (2012). Improvement of ITO-Free Inverted Polymer-Based Solar Cells by Using Colloidal Zinc Oxide Nanocrystals as Electron-Selective Buffer Layer. Org. Electron..

[25] Aizawa N., Pu Y., Watanabe M., Chiba T., Ideta K., Toyota N., Igarashi M., Suzuri Y., Sasabe H., Kido J. (2014). Solution-Processed Multilayer Small-Molecule Light-Emitting Devices with High-Efficiency White-Light Emission. Nat. Commun..

[26] Jia M., Xu X., Peng J., Zhang J., Yao C., Li L. (2016). Solution-processed Double-layer Electron-transport Layer for Conventional Blue Phosphorescent Organic Light-emitting Diodes. Adv. Opt. Mater..

[27] Ahn D., Lee S., Chung J., Park Y., Suh M. (2017). Impact of Interface Mixing on the Performance of Solution Processed Organic Light Emitting Diodes-Impedance and Ultraviolet Photoelectron Spectroscopy Study. ACS Appl. Mater. Interfaces.

[28] Amruth C., Szymański M., Łuszczyńska B., Ulański J. (2019). Inkjet Printing of Super Yellow: Ink Formulation, Film Optimization, OLEDs Fabrication, and Transient Electroluminescence. Sci. Rep..

[29] Van Dijken A., Meulenkamp E.A., Vanmaekelbergh D., Meijerink A. (2000). The Luminescence of Nanocrystalline ZnO Particles: The Mechanism of the Ultraviolet and Visible Emission. J. Lumin..

[30] Ma Y., Choi T.-W., Hang Cheung S., Cheng Y., Xu X., Xie Y.M., Li H.W., Li M., Luo H., Zhang W. (2019). Charge Transfer-Induced Photoluminescence in ZnO Nanoparticles. Nanoscale.

[31] Park S., Suh M., Kim K., Kim M., Cho H., Shin H., Seo H., Jung W., Jeon D. (2019). Effect of Spatial Molecular Configuration of ZnO/Polyethylenimine Hybrid Electron Injection Materials on OLEDs Performance. Org. Electron..

[32] Jung H., Kang S., Lee H., Yu Y.J., Jeong J.H., Song J., Jeon Y., Park J. (2018). High Efficiency and Long Lifetime of a Fluorescent Blue-Light Emitter Made of a Pyrene Core and Optimized Side Groups. ACS Appl. Mater. Interfaces.

[33] Takada M., Nagase T., Kobayashi T., Naito H. (2017). Electron Injection in Inverted Organic Light-Emitting Diodes with Poly (Ethyleneimine) Electron Injection Layers. Org. Electron..

[34] Qiu J., Weng B., Zhao L., Chang C., Shi Z., Li X., Kim H., Hwang Y. (2014). Synthesis and Characterization of Flower-Like Bundles of ZnO Nanosheets by a Surfactant-Free Hydrothermal Process. J. Nanomater..

