# Supplementary material for: Tailoring the Size and Shape of ZnO Nanoparticles for Enhanced Performance of OLED Device
Source: Nanomaterials (Basel). 2023 Oct 24;13(21):2816. doi: 10.3390/nano13212816 (PMC10647275; doi:10.3390/nano13212816)
Supplement: Supplementary file 1 [file nanomaterials-13-02816-s001.zip › nanomaterials-2652248-supplementary.pdf]

Supporting Information

Tailoring the Size and Shape of ZnO Nanoparticles for Enhanced Performance of OLED Devices

Nikita Khairnar †, Hyukmin Kwon †, Sangwook Park, Hayoon Lee, Jongwook Park *

Integrated Engineering, Department of Chemical Engineering, Kyung Hee University, Yongin-si 17104, Republic of Korea

*E-mail: jongpark@khu.ac.kr

† These authors contributed equally to this work.

1. Preparation

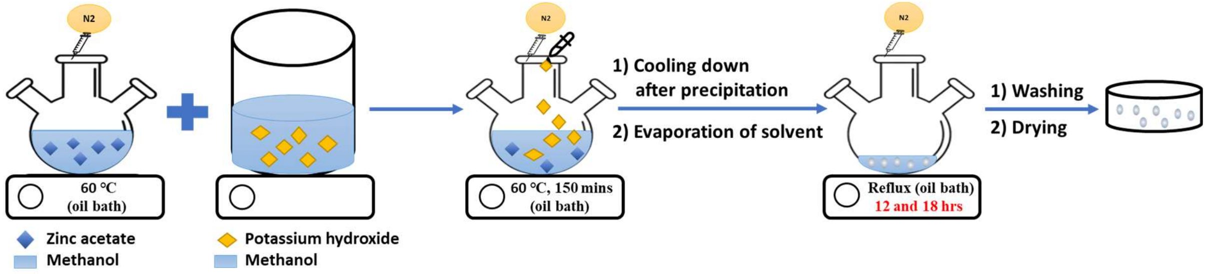

Figure S1. The schematic synthesis method for ZnO NPs

In order to obtain ZnO nanoparticles (NPs), we used zinc acetate and potassium hydroxide in methanol, following the procedure outlined in the literature [21]. Notably, we did not use any surfactants during the synthesis process because, from a thermodynamic point of view, surfactants such as trisodium citrate, ethylenediamine, poly(ethylene glycol), and cetyltrimethylammonium bromide can alter the surface free energy of different ZnO crystal faces and control the rates of nucleation and growth, potentially leading to their preferential growth or elimination [34]. While the ZnO-S and ZnO-I were purified, different reaction times were applied: 18 hours for ZnO-S and 12 hours for ZnO-I, respectively.

2. Crystallinity of ZnO

The crystallinity of ZnO is determined using the full width at half maximum (FWHM) of the (101) peak. The crystallite size is obtained by using the Scherrer equation for the (101) peak and is listed in Tables 1 and 2. a = $\lambda/\sqrt{3}$ sinθ; c = λ/sinθ; d = nλ/2sinθ; Vol= ($\sqrt{3}/2$) x a2c; D = kλ/βhkl cosθ D=Kλ/ (β cos θ) where D represents crystallite thickness, λ is the X-ray wavelength, K is the Scherrer constant (0.9), β is the full-width at half maximum, θ is the diffraction angle at the peak position. As time varies, the crystallite size increases from 5.90 nm to 9.92 nm, and the volume increases from 35.22 nm to 35.38 nm. This behavior is shown in Tables S1 and S2.

Table S1. Volume of unit cell of ZnO NPs.

Sample	2theta (°)	Theta (°)	Radians *180/π	Sin theta	a (Å)	c (Å)	FWHM (°)	Volume (nm)
ZnO-S	36.176	18.088	0.315695	0.310477	2.86357	4.96010	1.4814	35.22365
ZnO-I	36.123	18.061	0.315224	0.310029	2.867859	4.96728	0.88023	35.380574

Table S2. Crystallite size of ZnO NPs.

Sample	Peak position 2θ (°)	FWHM B size (°)	Crystallite size (nm)
ZnO-S	36.100	1.4814	5.90
ZnO-I	36.123	0.88023	9.92

3. Electroluminescence(EL) properties

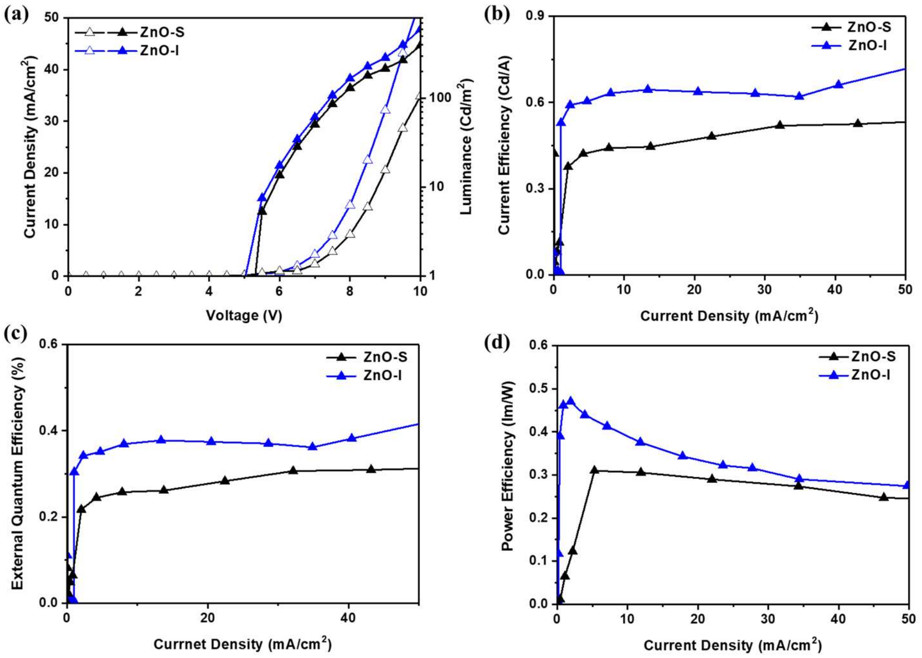

Figure S2. EL performance for blue device (a) current density(J)-voltage(V)-luminance(L) characteristics, (b) luminance efficiency(LE)-J curves, (c) external quantum efficiency(EQE)-J curves, (d) power efficiency(PE)-J curves.

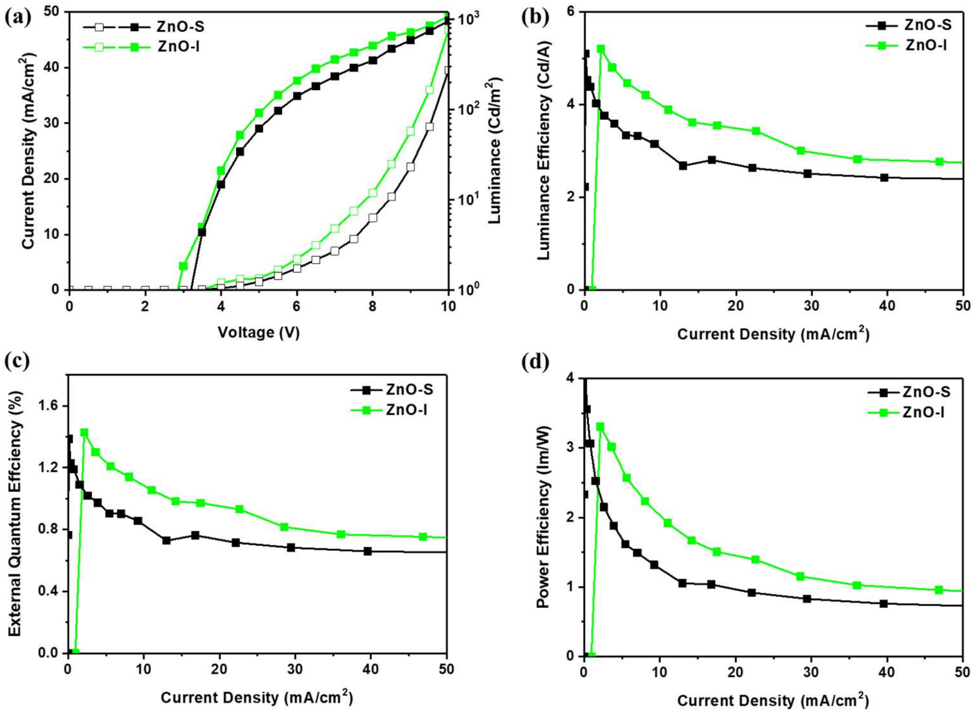

Figure S3. EL performance for green device (a) J-V-L characteristics, (b) LE-J curves, (c) EQE-J curves, (d) PE-J curves.

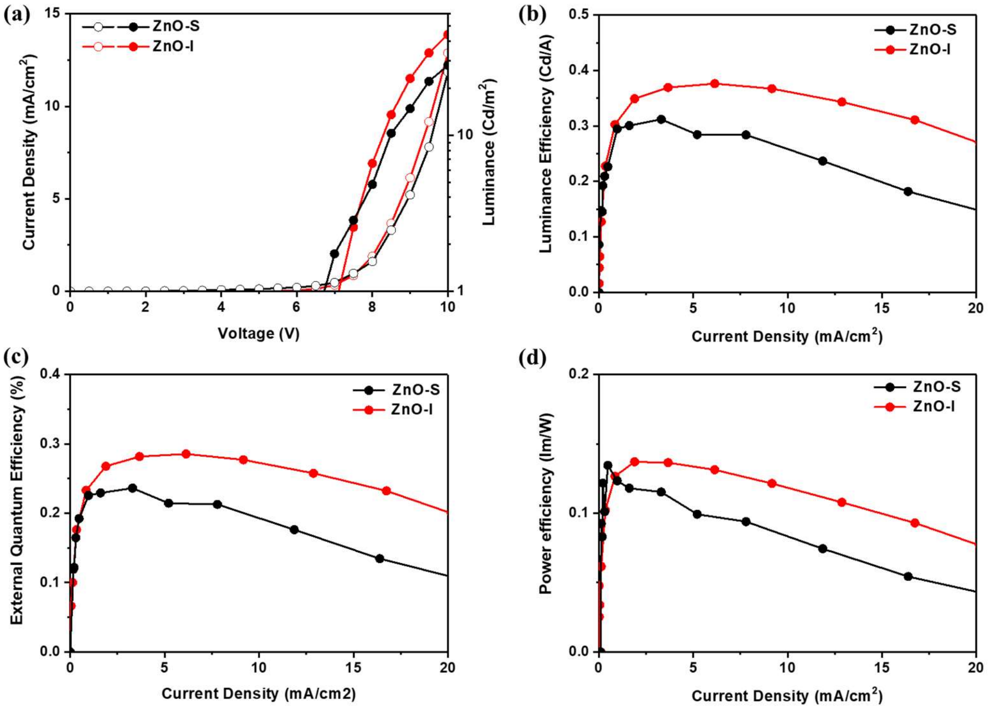

Figure S4. EL performance for red device (a) J-V-L characteristics, (b) LE-J curves, (c) EQE-J curves, (d) PE-J curves.

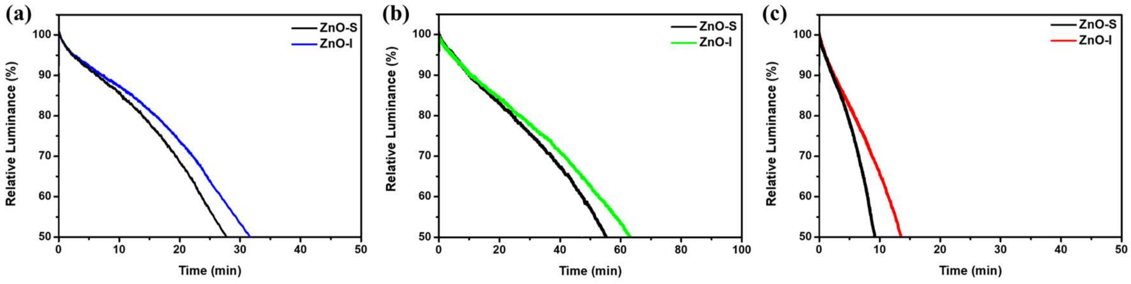

Figure S5. Lifetime of devices (a) blue, (b) green, (c) red.

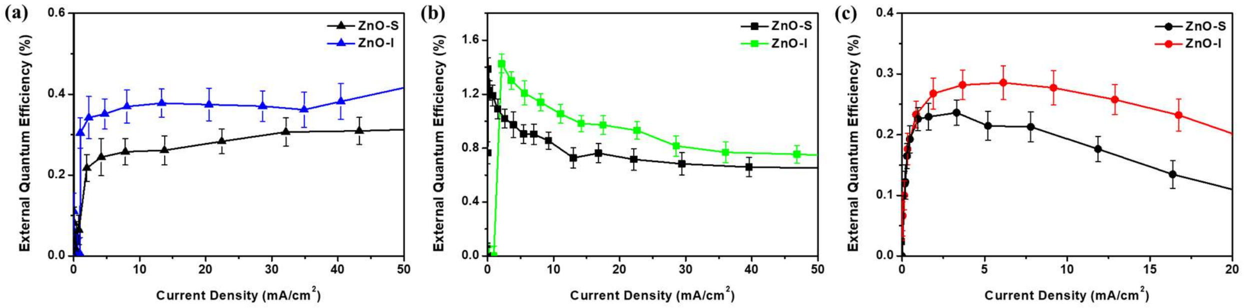

Figure S6. EQE curves according to the current density: (a) blue, (b) green, (c) red.
